# Analysis of single-nucleotide polymorphisms (SNPs) in human CYP3A4 and CYP3A5 genes: potential implications for the metabolism of HIV drugs

**DOI:** 10.1186/1471-2350-15-76

**Published:** 2014-07-02

**Authors:** Giulia Berno, Mauro Zaccarelli, Caterina Gori, Massimo Tempestilli, Andrea Antinori, Carlo Federico Perno, Leopoldo Paolo Pucillo, Roberta D’Arrigo

**Affiliations:** 1 Antiviral Drug Monitoring Unit, National Institute for Infectious Diseases “L. Spallanzani”, Rome, Italy; 2 Clinical Biochemistry and Pharmacology Laboratory, National Institute for Infectious Diseases “L. Spallanzani”, Via Portuense 292, Rome, 00149, Italy; 3 Clinical Department, National Institute for Infectious Diseases “L. Spallanzani”, Rome, Italy; 4 Department of Experimental Medicine and Surgery, University of Rome Tor Vergata, Rome, Italy; 5 Unit of Molecular Virology, Tor Vergata University Hospital, Rome, Italy

**Keywords:** Polymorphisms, Variability, Pharmacogenetics, Cytocrome P450

## Abstract

**Background:**

Drug metabolism via the cytochrome P450 (CYP450) system has emerged as an important determinant in the occurrence of several drug interactions (adverse drug reactions, reduced pharmacological effect, drug toxicities). In particular, CYP3A4 and CYP3A5 (interacting with more than 60% of licensed drugs) exhibit the most individual variations of gene expression, mostly caused by single nucleotide polymorphisms (SNPs) within the regulatory region of the CYP3A4 and CYP3A5 genes which might affect the level of enzyme production.

In this study, we sought to improve the performance of sensitive screening for CYP3A polymorphism detection in twenty HIV-1 infected patients undergoing lopinavir/ritonavir (LPV/r) monotherapy.

**Methods:**

The study was performed by an effective, easy and inexpensive home-made Polymerase Chain Reaction Direct Sequencing approach for analyzing CYP3A4 and CYP3A5 genes which can detect both reported and unreported genetic variants potentially associated with altered or decreased functions of CYP3A4 and CYP3A5 proteins. Proportions and tests of association were used.

**Results:**

Among the genetic variants considered, CYP3A4*1B (expression of altered function) was only found in 3 patients (15%) and CYP3A5*3 (expression of splicing defect) in 3 other patients (15%). CYP3A5*3 did not appear to be associated with decreased efficacy of LPV/r in any patient, since none of the patients carrying this variant showed virological rebound during LPV/r treatment or low levels of TDM. In contrast, low-level virological rebound was observed in one patient and a low TDM level was found in another; both were carrying CYP3A4*1B.

**Conclusions:**

Our method exhibited an overall efficiency of 100% (DNA amplification and sequencing in our group of patients). This may contribute to producing innovative results for better understanding the inter-genotypic variability in gene coding for CYP3A, and investigating SNPs as biological markers of individual response to drugs requiring metabolism via the cytochrome P450 system.

## Background

Genetic polymorphisms are well-recognized sources of individual differences in disease risk and treatment response. In fact, a high number of associations between human genetic variants and predisposition to adverse events have been described for different kinds of drugs, interacting with hundreds of proteins like receptors, transporters and metabolizing enzymes [1,2].

Cytochrome P450 (CYP450) is a family of isoenzymes responsible for the biotransformation of several drugs. Therefore, drug metabolism via the cytochrome P450 system has emerged as determinant in the occurrence of several drug interactions (drug toxicities, reduced pharmacological effect and adverse drug reactions) [3]. CYP3A4 and CYP3A5, representing 65% of isoforms in the cytochrome p450 enzyme family, interact with more than 60% of licensed drugs. Individual variations in single nucleotide polymorphisms (SNPs) within the regulatory genes of CYP3A4 and CYP3A5 might affect the level of enzyme production [4-6].

While the majority of SNPs have no biological consequences, a fraction of gene substitutions have functional significance and provide the basis for the diversity found among humans. So far, associations between human CYP3A4 and CYP3A5 genetic variants and predisposition to adverse drug reactions (ADRs) or therapy failure have often been hypothesized and described, mainly in HIV-infected patients treated with protease inhibitors (PI) whose metabolism is affected by induction or inhibition of CYP3A [7-11]. However, most studies published to date suffer from limitations, often represented by small study size or ethnic bias. This is probably due to the unavailability of robust, automated and inexpensive methods for detecting both known and unknown CYP3A genetic polymorphisms. Thus, the objective of this study was to develop and validate an effective, easy and inexpensive homemade Polymerase Chain Reaction Direct Sequencing (PCR-DS) approach for analyzing CYP3A4 and CYP3A5 genes, and detecting both reported and unreported genetic variants that may potentially be associated with altered or decreased functions of CYP3A4 and CYP3A5 proteins. Aiming to validate the method and avoid confounding drug - drug interactions, we included a homogeneous group of HIV patients undergoing steady treatment with RTV-boosted lopinavir (LPV/r) alone (LPV/r- monotherapy; pill dose: 200 mg lopinavir/50 mg Ritonavir, two pills BID).

## Methods

### Patients included and data collected

We analyzed 20 Caucasian HIV-infected patients undergoing treatment with LPV/r monotherapy with undetectable HIV-RNA (<40 copies/ml); all patients were in clinical follow-up at the Spallanzani Institute outpatient clinic. The ethics committee of the Lazzaro Spallanzani National Institute for Infectious Diseases review board approved the study and all patients gave written informed consent for inclusion. The patients’ characteristics are described in Table 1. In particular, virological rebound of HIV-RNA over the limit of detection (>40 copies/ml) and therapeutic drug monitoring (TDM) were used as indicators of treatment failure to evidence the possible reduction of LPV levels.

**Table 1:** Characteristics of analyzed patients

	
**Sex (male)**	13/20 (65%)
**Age (median years)**	46 (IQ range 44–50)
**CD4+ count (median cells/mm**^**3**^**)**	761 (IQ range 474–833)
**N. low level Virological Rebound**	5/20 (25%)
**LPV/r punctual TDM (median μg/ml)**	5.096 (IQ range 1.748–6.996)

### Genomic DNA isolation

In house methodologies based upon PCR and direct sequencing of CYP3A4 and CYP3A5 exons and flanking sequences (localized on human chromosome 7q21) were developed and optimized. Genomic DNA was extracted directly from 200 μl of whole blood using a commercially available kit (QIAamp DNA Mini Kit, Qiagen, Hilden, Germany) according to the manufacturer’s instruction.

### PCR amplifications

Primers for PCR were designed using the Primer-BLAST software developed at NCBI [12]. PCR primers were designed with a melting temperature between 57°C – 63°C (optimum at 60°C). The length of the primers was designed between 15–25 nucleotides with an optimum of 20 nts. The desired length of the PCR products was between 800–1500 bps. The primer had to have at least 2 total mismatches with unintended targets, including at least 2 mismatches within the last 5 bps at the 3′ end. The design of PCR primers in repeat regions was avoided. We selected homemade primers to amplify part of the 5′ upstream region of CYP3A4 [GenBank: D11131.1] and all exons with flanking intronic regions of both CYP3A4 [GenBank: AF209389.1] and CYP3A5 [GenBank: NG_007938.1]. The primer sets and PCR conditions used are shown in Table 2. The conditions for PCR amplification consist of an initial denaturation step at 93°C for 12 min followed by 40 cycles of 30 s of denaturation at 94°C, 30 s annealing at varying temperatures (refer to Table 2 for specific annealing temperatures), and a 2.30 min extension at 72°C with a final extension at 72°C for 10 min. The PCR reaction was carried out in 50 μl of solution using the following reaction mix: 5 μl of GeneAmp 10X PCR Buffer (Applied Biosystem, Foster City, CA, USA), 3 μl of 25 mM MgCl2 solution (Applied Biosystem), 33.95 μl of Dnase Rnase free water, 0.75 μl of each primer at a concentration of 10 μM, 0.8 μl of 10 mM solution of dNTPs and 0.75 μl of AmpliTaq Gold DNA Polymerase (5 u/μl, Applied Biosystems). See Table 2 for primer sequences and PCR annealing. In each PCR reaction, a negative control sample was included to ensure that no contamination of samples occurred during the analyses. The PCR products were electrophoresed on agarose gel to verify successful PCR and the absence of primer dimers. The products obtained were then subsequently used as templates for the sequencing reactions.

**Table 2:** Sequence and location of PCR and sequencing primers

**PCR name**	**Region**	**Primers sequence (forward and reverse)**	**Location of primers**	**PCR product size**	**PCR annealing temperature (°C)**
CYP3A4 gene					
A	5′ Proximal Region	A1: GGTCTGTCTGTCTGGGTATGC		296	61
		A2: CTCACCACACACTGACCTGCT			
B	EXON 1 (nt 1–71)	B1: AGAACCCAGAACCCTTTGGAC	1	1137	59
		B2: GTGCTCCTCTATCTGTGAGTA	78		
C	EXON 2 (nt 4004–4097)	C1: GCTCTCAGTGACCCTCTGTG	3532	1192	59
		C2: AACCCCTTTGTTCTGTCTCTCA	4723		
D	EXON 3 (nt 6009–6061)	D1: CCCTGGTGTCTGTACTTTCCA	5529	1200	59
		D2: TCCCAGCCTAGTTCAGACTGT	6728		
E	EXON 4 (nt 11502–11601)	E1: ATATCCACGTATGCACCACCC	11185	841	59
		E2: GAGCCACATGGAGACAGAGT	12025		
F	EXONS 5/6 (nt 13956–14069) (nt 14335−14423)	F1: CGACATCAGGGTCTCCTGAAC	13720	933	59
		F2: GATATGTAAACCCTGGCCCCT	14652		
G	EXON 7 (nt 15689–15837)	G1: CTGTTTGTCTGTCTTGACTGGA	15585	998	61
		G2: GCTGTTCAAGAAATAGTAGGTAGTC	16582		
H	EXON 8 (nt 16932–17059)	H1: TTGAGCTTCAGATTATGATTTGGG	16593	956	60
		H2: CTGGCTATCATGTGAGATGGC	17548		
I	EXON 9 (nt 17744–17810)	I1: AGCCATCTCACATGATAGCCA	17527	990	57
		I2: CTTGGTGGCTTGTAATTGACC	18516		
J	EXON 10 (nt 20166–20326)	J1: TGGGGGAGAGTACTACCTCATA	19785	952	60
		J2: AAGAGCCAATTCCTGTGTCCAT	20736		
K	EXON11 (nt 21912–22138)	K1: TTCCCGAATGCTTCCCACCT	21691	917	59
		K2: ATGCTACTGTACCGATGTAATGC	22607		
L	EXON 12 (nt 23198–23360)	L1: GGGGTGGCCCCTAAGTAAGA	23109	910	57
		L2: TTGGGTTGAAAAGGAGCCCA	24018		
M	EXON 13 (nt 25950–26502)	M1: TGACTCTTCAAAAACAGTTTGCCA	25518	1099	59
		M2: AGTTCTGACAAAGGCCCCAC	26502		
CYP3A5 gene					
N	EXON 1 (5001–5173)	N1: TAGAATGAAGGCAGCCATGGAG	4723	1032	60
		N2: GGGGATTTTCAGGGGCATGG	5774		
O	EXON 2 (8791–8884)	O1: GCTGGTTCTTCTGCACACAATC	8022	964	61
		O2: GAAACCTCAGAACTCCCTCCC	8985		
P	EXON 3 (10414–10466)	P1: ATGGAGAGTGGCATAGGAGAT	9878	1177	59
		P2: TGTGGTCCAAACAGGGAAGAGAT	11054		
Q	EXON 4 (12320–12419)	Q1: TGTCACCAGGTATCGAGGTCT	11062	1367	60
		Q2: GATGCTTACCCTTCGATTTGTGA	12428		
R	EXONS 5/6 (17934–18047) (18310–18398)	R1: CGCCCCACATACACTCAGAA	17746	1184	57
		R2: GGCTTGCTCTACACATAGCAT	18929		
S	EXON 7 (19685–19833)	S1: ATAGGGCCAGCTCCATCACTG	19492	1193	60
		S2: TTCTGAGTCTTTGGAGTGACCA	20684		
T	EXONS 8/9 (20904–21031) (22117–22183)	T1: GGCCTGAAAGAAGGGCAAAC	20750	1478	57
		T2: TCTTAGTGTCCCCGCCAGTA	22228		
U	EXON 10 (24340–24500)	U1: AGGATCATTCAAGGCACACACC	24009	1179	61
		U2: GCCTTGCTGCTGCCTTGCAG	25188		
V	EXON 11 (32220–32446)	V1: ACCTACCTATGATGCCGTGG	32225	880	57
		V2: GAGGACCTGTGCTGTCTTGT	33104		
W	EXON 12 (34767–34926)	W1: GCAGGATTTCAATGACCAGCC	34619	1173	59
		W2: CCCCCTGCCTGAATACACAC	35792		
Y	EXON 13 (36599–36809)	Y1: GGGTTCAACTGGGAAGGGTT	36118	1058	57
		Y2: GTGTGCAGGATGGCATCAGA	37175		

PCR reactions are named in alphabetical order. The same primers are used for PCR and sequencing reactions.

### DNA sequencing

The PCR products were purified with the PCR Cleanup KIT (Abbott, Wiesbaden, Germany) and then sequenced on both strands using the BigDye® Terminator Cycle Sequencing Kit v.3.1 (Applied Biosystems). The reaction mixture for the sequencing reaction contained 4 μl ABI PRISM Big Dye Terminator (Applied Biosystem), 3.8 μl water, 4 μl BigDye® TerminatorBuffer 5X1 (Applied Biosystems), 3.2 μl primer (1 pmol) and 5 μl of purified cDNA (40 ng), for a total volume of 20 μl. The sequencing conditions were: one cycle at 96°C for 3 min and 25 cycles (96°C for 30 s, 50°C for 10 s, 60°C for 4 min). Sequencing primers were the same of PCR reactions (see Table 2). The sequence products were purified by gel filtration chromatography using Sephadex G-50 resin (Sigma-Aldrich, Missouri, United States), in order to eliminate excess primers and/or unincorporated dideoxynucleotides (dNTPs), and then separated on an automated sequencer (ABI PRISM-3130 Genetic Analyzer, Applied Biosystems).

## Results

**Figure 1:**
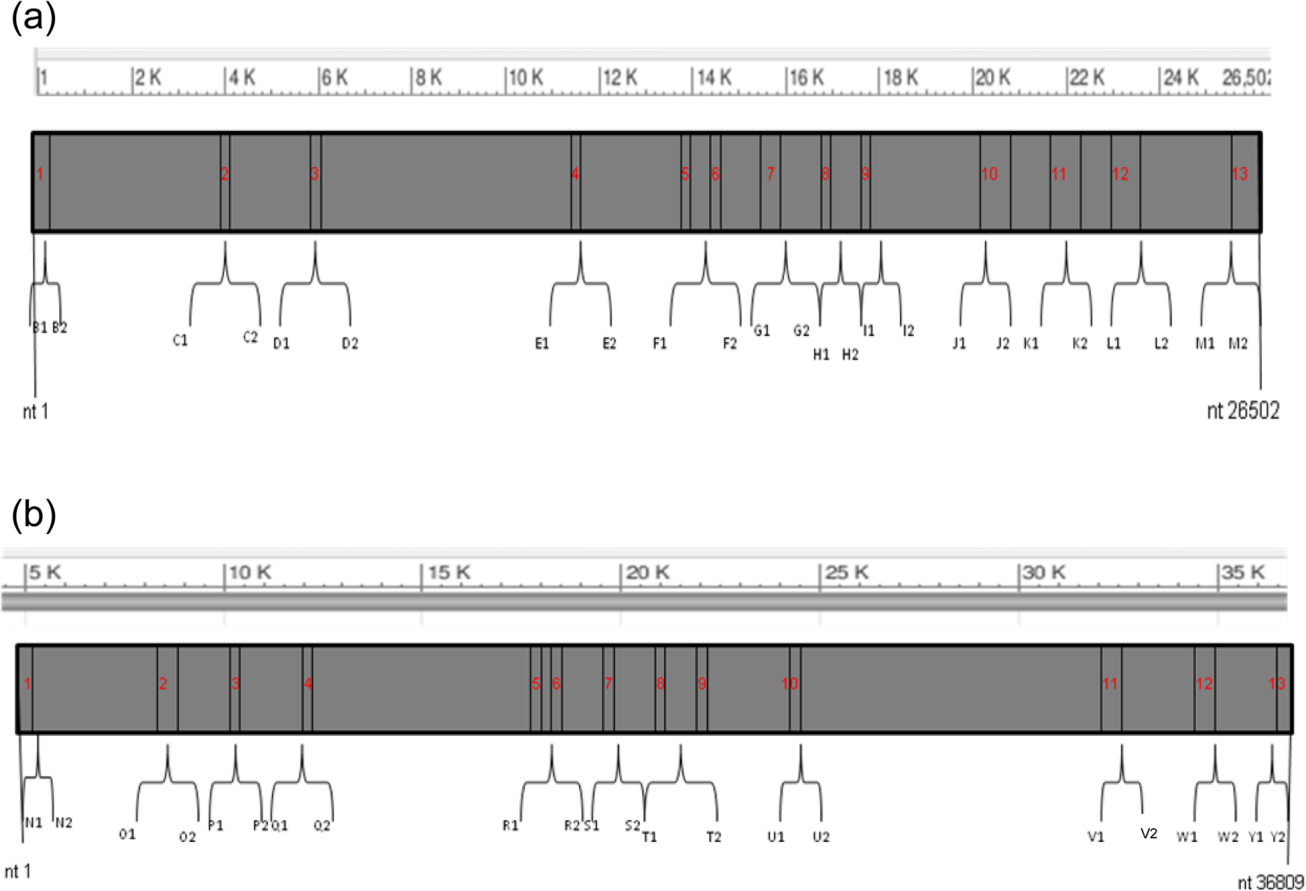
**Representative scheme of PCR and sequencing products. (a)** CYP3A4 gene. **(b)** CYP3A5 gene. In red are evidence all CYP3A4 and CYP3A5 exons. Alphabetical order is used for PCR primers.

By using SeqScape v.2.6 software we observed that all generated CYP3A4 and CYP3A5 DNA sequences matched the reference consensus sequences perfectly (see Figure 1). We initially focused our analysis on genetic variants significantly associated with altered or decreased protein expression (for **CYP3A4:** CYP3A4*1B, CYP3A4*2, CYP3A4*4, CYP3A4*5, CYP3A4*6, CYP3A4*8, CYP3A4*11, CYP3A4*12, CYP3A4*13, CYP3A4*16, CYP3A4*17, CYP3A4*18; for **CYP3A5**: CYP3A5*3, CYP3A5*5, CYP3A5*6, CYP3A5*8, CYP3A5*9, CYP3A5*10) in twenty HIV-1 infected patients undergoing LPV/r monotherapy, (Table 3) [4,12,13].

**Table 3:** Frequency of CYP3A4 and CYP3A5 polymorphisms in 20 HIV-infected patients

**Genotype/20 pts**			
**Gene**	**Polymorphism**	**wt ( *n. pts *)**	**m ( *n. pts *)**
**CYP3A4**			
	CYP3A4*1B; 816 A > G 5′-flanking region	17/20	3
	CYP3A4*2; 15831 T < C exon 7	20	
	CYP3A4*4; 13989 A > G exon 5	20	
	CYP3A4*5; 15820 C > G exon 7	20	
	CYP3A4*6; 17775 *insA* exon 9	20	
	CYP3A4*8; 14026 G > A exon 5	20	
	CYP3A4*11; 21973 C > T exon 11	20	
	CYP3A4*12; 22002 C > T exon 11	20	
	CYP3A4*13; 22132 C > T exon 11	20	
	CYP3A4*16; 15721 C > G exon 7	20	
	CYP3A4*17; 15733 T > C exon 7	20	
	CYP3A4*18; 20178 T > C exon 10	20	
**CYP3A5**			
	CYP3A5*3; 12083 G > A intron 3	17/20	3
	CYP3A5*5; 18049 T > C intron 5	20	
	CYP3A5*6; 19787 G > A exon 7	20	
	CYP3A5*8; 8801 C > T exon 2	20	
	CYP3A5*9; 24483 G > A exon 10	20	
	CYP3A5*10; 34850 T > C exon 12	20	

Wt: wild-type, m: mutated.

Patients started LPV/r monotherapy after achieving viral suppression (Table 1). Six patients interrupted LPV/r for viral rebound after a median time of 7 months.

Among the genetic variants considered, CYP3A4*1B (expression of altered function) was only found in three patients (15%) and CYP3A5*3 (expression of splicing defect) in three other patients (15%) by proportions and tests of association [8,12,14,15]. CYP3A5*3 did not appear to be associated with decreased efficacy of LPV/r in any of the patients. Indeed, none of the patients carrying this variant showed virological rebound during LPV/r treatment or low level of TDM. In contrast, low-level virological rebound was observed in one patient and a low TDM level (1.748 μg/ml vs. a mean of 4.719, IQ range 3.008-6.996) in a second patient; both were carrying CYP3A4*1B.Moreover, many previously unreported SNPs were observed, and data collection in a dedicated database is in progress. Therefore, our PCR-DS method is able to detect clinically relevant substitutions occurring in CYP3A4 and CYP3A5 without using commercial kits, and with a considerable reduction of costs. In addition, the PCR primers were also designed to cover a large part of the flanking intronic sequence (see Figure 1). Indeed, by displaying the entire gene sequence it is possible to investigate new polymorphisms which are potentially able to modulate CYP3A gene expression and enzyme activity, and thus affect drug metabolism.

Our protocols showed an overall efficiency of 100% in terms of DNA amplification and sequencing. Primers were selected with similar or identical annealing temperatures to perform the screening of several exons in the course of one analysis in order to reduce the number of PCR machines involved in the process and save laboratory time. No primer interactions (namely primer dimers) were observed.

## Discussion

The ability of even a single cytochrome P450 isoenzyme to metabolize many drugs may be responsible for drug-drug interactions which may possibly lead to ADRs that occur in clinical practice. This effect reflects the possibility that other active compounds can activate or inhibit a particular isoenzyme, altering the biotransformation of substrates. The inhibition of an enzyme that performs a catabolic activity on a second one causes an increase in plasma levels of the second, therefore prolonging its action. The consequences can be particularly severe if the inhibited enzyme is solely or primarily responsible for the biotransformation of the drug. The inhibition of the metabolism of a drug can also result in treatment failure if its effectiveness resides in one or more metabolites [1,3,4,6]. A typical example is the association of LPV/r: Ritonavir competes with Lopinavir for the same cytochrome P450 (CYP3A) isoenzyme but has a greater affinity, thus acting as a “booster”, increasing the plasma half-life of LPV [16,17].

To date, the scientific community has paid great attention to the issue of human genetic variability in drug-treated patients and its role in the occurrence of adverse drug reaction (ADR), but little information is currently available regarding whether CYP3A human gene expression profiles are predictive of adverse drug reactions (ADRs). Despite the fact that more than 60% of all licensed drugs interact with CYP3A, the real significance of CYP3A SNPs has been only partially elucidated and few, limited studies have been performed. In this light, identifying mutations that can act as sentinels of ADR occurrence is crucial for improving the criteria for the selection of drugs administered to single patients. This is even more relevant from a practical point of view, since the methods for detecting SNPs in CYP3A sequence are used solely for research purposes.

In this study, we sought to improve the performance of a sensitive screening for CYP3A polymorphism detection. The novelty of this method relies on the improvement of data accessibility by sequencing CYP3A4 and CYP3A5 genes in a faster and less expensive way than the traditional or commercially available methods.

Primarily, we reviewed the literature in similar population based studies on the available methods for detecting SNPs in the CYP3A gene. Real Time PCR or restriction fragment length polymorphism (RFLP) were the most used, providing analyses of only defined and small CYP3A genomic areas, but lacked investigation of SNPs which are not yet described [4,18,19]. Hence, we developed a simple, inexpensive and reliable method based on classical qualitative PCR and subsequent sequencing of CYP3A (A4; A5), including all exons and part of the intron region (see Figure 1). This method, based on the sequence screening of CYP3A4 and CYP3A5 genes may provide rapid, effective and comprehensive information on the molecular analysis of CYP3A4 and CYP3A5 human variability.

The entire analysis, including the DNA purification, preparation of the reaction mixture and PCR running takes 9 hours. None of the reagents used in the PCR interfere with the sequencing reaction; therefore, it is possible to perform sequencing directly from PCR products. Its application, aimed at optimizing diagnostic-therapeutic strategies for managing different drugs in clinical practice, may be useful for elucidating the genetic determinants of the pathogenetic mechanisms related to ADR occurrence and the role of unreported CYP3A SNPs.

Analysis of genes deputed to the metabolism of drugs, when applied to patients who have shown ADRs, may be the most comprehensive method for highlighting the point mutation(s) that caused the reactions. In particular, identifying CYP3A SNPs may also be helpful when selecting the most appropriate antiretrovirals (PI used in HIV therapy) to be included in the first line regimen of HIV-1 infected patients. They may contribute to minimizing HIV resistance, reducing the general cost of antiretroviral therapy and avoiding ADR occurrence [7,20,21].

Among twenty HIV-positive patients undergoing LPV/r monotherapy enrolled in this study, CYP3A4*1B was found in two patients with treatment failure and decreased TDM of LPV. These data may suggest that the presence of CYP3A4 SNPs, in particular the CYP3A4*1B variant, may have a role in reducing LPV efficacy. However, this was a pilot analysis, therefore the method should be evaluated in a larger set of patients.

In addition, our method is widely applicable to all therapeutic regimens that include drugs metabolized by CYP3A. Discovering new SNPs which can determine the onset of ADR or affect treatment efficacy will allow for the use of personalized therapeutic regimens that are free from side effects. For example, immunosuppressant drugs such as Tacrolimus (currently used in the management of solid organ transplant recipients) are also extensively metabolized by CYP3A isoenzymes [22-25]. In this context, our study may also be useful for further analyzing SNPs with significant influence on the metabolism and/or inter-individual pharmacokinetic variability of Tacrolimus in tranplants.

## Conclusions

Here we describe the development of a sequence screening method based on primer extension (with no additional secondary steps as nested PCR) for rapid detection of SNPs in the CYP3A4 and CYP3A5 gene sequence by studying samples from patients undergoing therapy with antiretroviral drugs metabolized via the CYP450 family.

Our method, which exhibited an overall efficiency of 100% (DNA amplification and sequencing in our group of patients), may contribute to producing innovative results for better understanding the inter-genotypic variability in gene coding for CYP3A and investigating SNPs as biological markers of individual response to drugs requiring metabolism via the cytochrome P450 system.

## Competing interests

The authors declare that they have no competing interests.

## Authors’ contributions

GB contributed to developing the method for detecting CYP450 SNPs, prepared the DNA samples and performed all molecular analyses. MZ participated in sample collection, drafting, data analysis and the critical revision of the manuscript for important intellectual content. CG and MT helped coordinate and draft the manuscript. AA revised the manuscript. CFP helped draft the manuscript, revised it critically for important intellectual content and gave the final approval of the version to be published. LPP helped draft the manuscript and revised it critically for important intellectual content. RD designed the study, supervised all the work and drafted the manuscript. All authors read and approved the final manuscript.

## Pre-publication history

The pre-publication history for this paper can be accessed here:

http://www.biomedcentral.com/1471-2350/15/76/prepub
